# High school teachers’ awareness of anxiety and depression in adolescents: cross-country analysis

**DOI:** 10.1192/bji.2025.17

**Published:** 2026-02

**Authors:** Nazish Imran, David M. Ndetei, Rodrigo Nel Cordoba Rojas, Afzal Javed

**Affiliations:** 1 Professor of Child & Adolescent Psychiatry, Child and Family Psychiatry Department, King Edward Medical University/Mayo Hospital, Lahore, Pakistan. Email: nazishimrandr@gmail.com; 2 Professor of Psychiatry, Africa Mental Health Research and Training Foundation, Nairobi, Kenya; 3 Auxilliary Professor of Career, University of Rosario Center for Mental Health (CeRSaMe), School of Medicine and Health Sciences, University of Rosario, Bogota, Colombia; 4 Professor of Psychiatry, Pakistan Psychiatric Research Centre, Fountain House Institute for Mental Health, Lahore, Pakistan

**Keywords:** Adolescents, school teachers, mental health literacy, anxiety, depression

## Abstract

**Background:**

Schools are a crucial part of child and adolescent care systems, especially in low- and middle-income countries. In today’s complex and rapidly evolving educational landscape, the role of high school teachers extends far beyond delivering academic content. School teachers are in a good position to identify common mental health problems in adolescents. However, their mental health literacy levels remain unclear.

**Aims:**

To evaluate high school teachers’ mental health literacy about anxiety and depression and its determinants in three countries (Kenya, Pakistan and Colombia).

**Method:**

A self-administered questionnaire comprising the Anxiety Literacy Questionnaire (A-Lit), Depression Literacy Questionnaire (D-Lit) and statements from the teachers’ quiz in the Mental Health and High School Curriculum Guide was used to collect data.

**Results:**

We received 748 responses from teachers in the three countries; 56.6% of respondents identified as females. Mean scores on the A-Lit and D-Lit were low: 9.14 (s.d. = 3.14) and 9.36 (s.d. = 3.10) respectively (maximum score: 22 on each instrument). Many statements on the Mental Health and High School Curriculum Guide also had low proportions of correct answers. Country of residence (Colombia) and prior training in child mental health were positively correlated with total scores on the D-Lit (*P* < 0.05). Only 30.3% of teachers had confidence in helping students with anxiety and depression.

**Conclusions:**

The participating high school teachers had low mental health literacy about anxiety and depression. By using teacher training and awareness programmes in schools, policymakers could work towards creating a more supportive and informed environment for students facing mental health challenges.

Adolescence is a crucial period for mental health, as young people undergo significant physical, emotional, cognitive and social changes. During this time, they may experience intense emotions, stress and pressure from various sources, including academic expectations, peer relationships and personal identity formation. It is also a time when mental health problems such as anxiety, depression and non-suicidal self-injury often emerge.^1^ A meta-analysis of 29 studies involving a total of 80 879 children and adolescents worldwide found that the pooled prevalence estimates of clinically elevated anxiety and depression during the COVID-19 pandemic were 20.5 and 25.2% respectively.^2^

High school teachers play a crucial role in supporting adolescents’ mental health and well-being, given the significant time students spend in school.^3^ To effectively recognise and respond to mental health problems, teachers need a comprehensive understanding of mental disorders.^4^ However, educators juggle numerous responsibilities, and their prioritisation of mental health literacy might not be as high as necessary. Studies also suggest that teachers attribute their ignorance of mental health problems to their heavy workloads and other commitments.^5^ However, the role of schools and teachers in adolescent mental health cannot be overstated. Understanding risk factors and causes, identifying and managing mental health problems, and promoting behaviours that encourage seeking help are all included in mental health literacy. Unfortunately, despite the increasing rates of internalising disorders among adolescents, research has shown that high school teachers in various regions, including Europe, the USA, Asia and Africa, often lack robust knowledge about mental health problems and feel uncertain about aiding students who suffer from them.^4,6^ In a study conducted in Kenya, teachers recognised learning difficulties (intellectual disability and specific problems such as dyslexia), externalising and internalising problems, bizarre behaviour and problem substance use among students but reported lack of skills and time as the main challenges in dealing with students’ mental health problems.^7^ A study from Pakistan highlighted the necessity of specialised training programmes to improve mental health literacy among teachers, particularly in locations with limited resources.^8^ With the right information and resources, teachers can contribute to creating a school environment that promotes mental health and helps students thrive academically and personally. Therefore, the present study aimed to estimate and evaluate levels of knowledge and attitudes regarding anxiety and depression held by a sample of high school teachers in three countries (Colombia, Kenya and Pakistan) and to investigate any potential associations between a few chosen sociodemographic variables. In addition, we looked at possibilities to pinpoint areas in which teachers may be deficient in fundamental understanding of mental illnesses and in which they might exhibit prejudice towards students with these illnesses, in order to develop teacher training programmes.

## Method

The proposed study was conducted in selected schools in three countries (Pakistan, Kenya and Colombia). Initially, seven countries were invited to participate, but only these three agreed to take part and were therefore included. The selection of schools was based on feasibility, taking into account the limited resources and funding available for the study. School selection was also influenced by the willingness of administrators to participate, facilitate access to teachers and support the project’s objectives, particularly in training teachers to enhance their mental health literacy. All the high school teachers in the participating schools were provided information about the study and those who voluntarily agreed to participate were chosen for the study. There was no compulsion to participate. Exclusion criteria include lack of informed consent and teachers who had not engaged in active teaching during the previous 6 months.

### Ethical approval

The study was part of an initiative by the World Psychiatric Association (WPA), which gave formal ethical approval for the project (23 April 2023). Ethical approval was also obtained from institutional review boards in the three participating countries: the Institutional Review Board of King Edward Medical University (approval no: 110/RC/KEMU); Kenyatta University’s Ethics Research Committee (approval no. PKU/2627/E1752); and the National Commission for Science, Technology, and Innovation (NACOSTI) (license number NACOSTI/P/23/23559). Schools and teachers were asked to participate in the study after being informed about the research. The study was conducted according to the Helsinki Declaration of 1975, as revised in 2008, and written informed consent was obtained from all participants.

### Procedure and participants

Following written informed consent, a self-administered questionnaire was used to assess teachers’ mental health literacy. It was in the English language and comprised the following parts.

#### Demographic variables

Demographic information about the teachers was collected, including age, gender, academic degree, length of teaching experience and prior participation in mental health seminars.

#### Knowledge about anxiety and depression

The next section of the questionnaire asked questions about anxiety and depression. The Anxiety Literacy Questionnaire (A-Lit) and Depression Literacy Questionnaire (D-Lit) are scales used to assess mental health literacy specific to anxiety and depression.^9–11^ Both are 22-item tools, answered with ‘true’, ‘false’ or ‘don’t know’. Sample items include ‘People with anxiety disorder often speak in a rambling and disjointed way’ and ‘Being easily fatigued may be a symptom of anxiety disorder’. Each correct response receives one point and all items are summed to produce a total score (range 0–22). Higher scores indicate higher literacy regarding anxiety or depression. The scales have been used in community settings.^12–13^ In the current study the A-Lit and D-Lit both had a Cronbach’s alpha of 0.7. This section also included 17 questions related to anxiety and depression from the quiz in the Mental Health and High School Curriculum Guide, which were answered ‘yes’ or ‘no’.^14^

The last part of questionnaire asked about confidence in providing help. Teachers were asked ‘How confident do you feel in helping a student with a mental health problem?’ (answered on a 5-point Likert scale: ‘not at all’, ‘a little bit’, ‘moderately’, ‘quite a bit’ and ‘extremely’).^15^

Data entry and analyses were completed using the SPSS 26 statistical package for Windows. Descriptive statistics were computed for the teachers and responses to questionnaires were evaluated. Independent samples *t*-tests and analysis of variance (ANOVA) were used to examine differences in knowledge and attitudes scores between participants as per gender and country. To explore the determinants of the teachers’ knowledge about anxiety and depression, normality of the data was checked using the Kolmogorov–Smirnov test. Since the data were not normally distributed, Spearman’s correlation coefficient was computed. Medians of anxiety and depression literacy scores in the three countries were compared using the Kruskal–Wallis test. Multiple comparison between countries was computed if significant group differences in median scores were found. The level of significance was set at α = 0.05.

## Results

Table 1 shows participants’ demographic data. The sample (*n* = 748 high school teachers) was predominantly female (56.6%) and most had Bachelor’s qualification (56.6%). Approximately 60% were from Kenya, 31% from Pakistan and 8% from Colombia. The number of years participants had been teaching ranged from 1 to 35 years (mean 10.98, s.d. = 8.67). When asked about sources of previous knowledge about mental health, 18% reported that they had read and learned about mental health on their own, and 32.2 % had attended seminars related to mental health.

**Table 1:** Participants’ demographic data (*n* = 748 high school teachers)

Characteristics	*n* (%)
Country	
Kenya	450 (60.2)
Pakistan	236 (31.6)
Colombia	62 (8.3)
Age, years: mean (s.d.)	36.98 (9.65)
Gender, *n* (%)	
Female	423 (56.6)
Male	325 (43.4)
Academic degree, *n* (%)	
Intermediate	11 (1.5)
Bachelor’s	423 (56.6)
Master’s	249 (33.3)
Higher qualification	65 (8.7)
Teaching experience, years: mean (s.d.)	10.98 (8.67)
Length of time working in current school, years: mean (s.d.)	6.53 (6.45)
Previous attendance of mental health seminars, *n* (%)	
Yes	241 (32.2)
No	507 (67.7)

Table 2 shows the teachers’ mean literacy scores regarding anxiety and depression in the three countries. There were no statistically significant differences between genders regarding anxiety and depression literacy in the three countries.

**Table 2:** High school teachers’ knowledge about anxiety and depression

Assessment scales	Total sample (*n* = 748), mean (s.d.)	Kenya (*n* = 450), mean (s.d.)	Pakistan (*n* = 236), mean (s.d.)	Colombia (*n* = 62), mean (s.d.)
Anxiety Literacy Questionnaire total score (max. possible score 22)	9.14 (3.14)	9.13 (3.15)	9.17 (2.87)	9.05 (3.99)
Depression Literacy Questionnaire total score (max. possible score 22)	9.36 (3.10)	9.47 (3.05)	8.84 (2.66)	10.80 (4.40)
Teachers’ quiz in Mental Health and High School Curriculum Guide (max. possible score 17)	9.19 (1.61)	9.49 (1.56)	8.65 (1.62)	9.22 (1.36)

Regarding specific questions about emotional problems (from the teachers’ quiz in the Mental Health and High School Curriculum Guide) many items had low proportions of correct answers. The three items with the lowest proportions of correct answers were: ‘Three of the strongest risk factors for teen suicide are: romantic break-up, conflict with parents and school failure’ (false; 7.4% answered correctly); ‘Diet, exercise and establishing a regular sleep cycle are all effective treatments for many mental disorders in teenagers’ (false; 14.1%); and ‘Generalised anxiety disorder usually arises from being burned out by stressful events’ (false; 18.8%) (Table 3).

**Table 3:** High school teachers’ knowledge about mental illnesses (emotional problems)^a^

Item	Correct answer	Proportion of correct responses, %
A phobia is an intense fear of something that might be harmful (such as heights, snakes)	True	94
Useful interventions for adolescent mental disorders include both psychological and pharmacological treatment	True	86.9
Mental distress can occur in someone who has a mental disorder	True	81.9
Stigma against the mentally ill is uncommon in [the country’s name]	False	65
The stresses of being a teenager are a major factor leading to adolescent suicide	False	24.4
Three of the strongest risk factors for teen suicide are: romantic break-up, conflict with parents and school failure	False	7.4
A depressed mood that includes a drop in school grades and lasts for a month or longer in a teenager is very common and should not be confused with clinical depression that may require professional help	False	20.4
Generalised anxiety disorder usually arises from being burned out by stressful events	False	18.8
Diet, exercise and establishing a regular sleep cycle are all effective treatments for many mental disorders in teenagers	False	14.1
Bipolar disorder is another name for manic depressive illness	True	70.9
The panic attacks that occur as part of panic disorder usually come ‘out of the blue’	True	57.6
Youths who have social anxiety disorder do not get well with treatment	False	62.6
A psychiatrist is a medical doctor who specialises in treating people who have a mental illness	True	88.3
Panic disorder is a type of anxiety disorder	True	88.4
Lack of pleasure, hopelessness and fatigue can all be symptoms of clinical depression	True	83.9
Mental disorders are psychological problems that are often caused by poor nutrition	False	57.2

a. Items are taken from the teachers’ quiz in the Mental Health and High School Curriculum Guide.

### Associations between demographic variables and knowledge about anxiety and depression

In our exploration of the determinants of teachers’ knowledge about anxiety and depression, we employed Spearman’s correlation coefficient (ρ). We found that literacy about depression and about anxiety were correlated (ρ = 0.545; *P* < 0.001); total depression literacy was negatively correlated with Pakistan (ρ = −0.135 (*P* < 0.001) and positively correlated with Colombia (ρ = 0.137; *P* < 0.001); years of teaching experience was negatively correlated with total depression literacy (ρ = 0.082; *P* < 0.05); and prior training was positively correlated with depression literacy (ρ = 0.084; *P* < 0.05) and anxiety literacy (ρ = 0.139; *P* < 0.001). The Kruskal–Wallis test showed a non-significant difference between the countries in anxiety literacy (*z* = 0.062; d.f. = 2; *P* = 0.97) and a significant difference between the countries in depression literacy (*z* = 21.341; d.f. = 2; *P* < 0.001). The mean rank of the total depression literacy score for each country was 441.22 for Colombia, 358.96 for Kenya and 311.80 for Pakistan. Multiple pairwise analysis of depression literacy scores between the countries is reported in Table 4.

**Table 4:** Pairwise comparison of countries on Depression Literacy Questionnaire total scores

Comparison	Test statistic	s.e.	Std test statistic	*P*	Adj. *P*
Pakistan–Kenya	−46.755	16.405	−2.850	0.004	0.013
Pakistan–Colombia	−129.416	29.564	−4.377	0.000	0.000
Kenya–Colombia	−82.661	28.339	−2.917	0.004	0.011

Std, standardised; Adj., adjusted.

## Discussion

To the best of our knowledge, this is the first study to investigate knowledge about anxiety and depression held by high school teachers in Kenya, Pakistan and Colombia. Understanding teachers’ perceptions and awareness is crucial, as schools play an integral role in adolescent mental health support and intervention.^16^ Our study revealed that teachers had limited knowledge about mental health. Most teachers were not aware of the common symptoms of mental illnesses or of basic treatments. In addition, only few teachers felt confident in helping students struggling with mental health problems. This lack of knowledge, low recognition and low confidence may lead to difficulty/inability in supporting students who have mental health problems. It also raises questions about existing support systems and resources available to students who are struggling with anxiety and depression.

Mental disorders such as anxiety, depression and non-suicidal self-injury are prevalent among adolescents and can have significant negative impacts on their overall well-being and academic performance.^17^ Adolescents spend considerable time with their teachers, who are likely to know them well because of their regular interaction over extended periods. It is therefore crucial for teachers to have a basic understanding of these mental health problems in order to provide appropriate support and intervention for their students.^3^ Most of the participants in our study did not correctly identify the symptoms of anxiety and depression, believing, for example, that people with depression and anxiety speak in a rambling way and that hearing voices may be a common sign of these illnesses. This finding indicates that there is an urgent need to work with teachers and teaching professionals to help them understand common mental illnesses.

Regarding the treatment of mental illnesses in adolescence, most teachers believed that antidepressants are addictive and that they start working straight away and that acupuncture is as effective as cognitive–behavioural therapy for depression. The majority of teachers also seemed unaware of risk factors for mental illnesses and suicide. This finding is consistent with earlier research indicating gaps in teachers’ preparedness and training in recognising and supporting students’ mental health needs.^18^ A study on mental health literacy among Japanese high school teachers revealed that they generally had low correct recognition rates for major mental illnesses, and relatively few (19%) felt confident in their ability to help students with depressive symptoms.^6^ Teachers often feel ill-prepared to support students with mental health problems because of their lack of information and skills in this area. Studies suggest an association between less stigmatising views with better knowledge of depression and suicide and more favourable attitudes towards prevention efforts.^19^

Our study highlights the importance of sociodemographic factors in understanding teachers’ knowledge and attitudes towards mental health problems. Findings of differences in literacy based on country of residence highlight the potential impact that cultural, educational and systemic factors within different countries can have on mental health literacy. We found no statistically significant differences between genders regarding anxiety and depression literacy, perceived knowledge and information across the three countries studied. Yamaguchi et al noted that correct recognition of specific mental illnesses was significantly lower among male teachers compared with female teachers.^6^ In general, although some studies have found gender differences in the recognition of and literacy about mental disorders like anxiety and depression,^6,20,21^ such differences are not always consistent across different populations or settings. The fact that prior attendance of child mental health seminars specifically addressing anxiety and depression improved knowledge scores in those areas in the current study points to the effectiveness of targeted educational interventions in increasing mental health literacy.^22–23^ This is also in line with previous studies suggesting a significant association between improved levels of knowledge about mental illnesses and how to deal with students who have mental health problems among teachers who participate in mental health training programmes.^24^ It underscores the importance of such training for educators, as it equips them with the knowledge necessary to address these problems in students.^25–26^ Both teacher training in mental health literacy and standardised screening tools have benefits in early detection of student mental health problems. Since teacher training equips educators to recognise problems and offer assistance without the need for more funding, it is more economical and sustainable. On the other hand, screening tools provide organised evaluations but necessitate budgetary outlay and skilled staff. It may be helpful to combine screening tools for focused assessments with empowering teachers through training to support students’ well-being.

### Limitations and strengths

The current study has several limitations. It focuses on high school teachers’ knowledge related to anxiety and depression in three selected countries, with uneven sample distribution, which may limit the applicability of findings to other teacher groups as well as generalisability to other geographical areas or countries. Also, although the tools in the study are widely used to assess literacy about depression and anxiety and have shown reliability in study settings they have not been validated in these countries. Another limitation of this study is the potential for cultural biases in self-reported data, particularly given their cross-country nature. Self-administered questionnaires may be subject to bias as participants may not always provide accurate information because of a desire to conform to social norms or because they misunderstand the questions. The cross-sectional nature of the study means it can establish correlation but not causation. It captures knowledge at a single point in time, not allowing for the assessment of changes over time. The study also did not explore the reasons behind the low levels of mental health literacy among teachers, such as potential barriers or challenges they may face in addressing students’ mental health problems. Despite these limitations, the diverse sample from three countries and the large sample size might increase the reliability of the results. Furthermore, association of various demographic factors with knowledge about common mental illnesses provides a more comprehensive understanding of the issues around mental health literacy.

### Implications

Our study can inform policy and practice in high school settings so that teachers are able to identify adolescents at risk of mental illness and steer them towards the right type of help. Comprehensive strategies are required to improve teachers’ knowledge and attitudes regarding common mental disorders among adolescents. By understanding mental health problems and the potential impact they can have on students, high school teachers can play a vital role in mental health promotion and illness prevention efforts in schools.

## Data Availability

The data that support the findings of this study are available from the corresponding author (N.I.) on request.
